# The impact of COVID-19 pandemic on the world’s major economies: based on a multi-country and multi-sector CGE model

**DOI:** 10.3389/fpubh.2024.1338677

**Published:** 2024-03-19

**Authors:** Mingsong Sun, Shiling Yan, Tingting Cao, Jingwen Zhang

**Affiliations:** ^1^School of Economics and Finance, Huaqiao University, Quanzhou, China; ^2^Department of Agriculture and Forestry Technology, Weifang Engineering Vocational College, Qingzhou, China; ^3^Department of Economics and Management, Weifang University of Science and Technology, Shouguang, China; ^4^Postdoctoral Station, Dongbei University of Finance & Economics, Dalian, China; ^5^Acupuncture and Tuina School, Chengdu University of Traditional Chinese Medicine, Chengdu, China

**Keywords:** COVID-19 pandemic, public health, economic impact, CGE model, multi-country analysis, policy interventions

## Abstract

**Objective:**

To quantitatively assess the impact of COVID-19 pandemic on public health, as well as its economic and social consequences in major economies, which is an international public health concern. The objective is to provide a scientific basis for policy interventions.

**Subject and methods:**

This study utilizes a multi-country, multi-sector CGE-COVID-19 model to analyze the repercussions of the pandemic in 2022. The re-search focuses on quantifying the effects of COVID-19 on the macroeconomy and various industry sectors within six economies: the United States, China, the EU, the United Kingdom, Japan, and South Korea.

**Results:**

The COVID-19 pandemic shock had the most significant impact on China and the EU, followed by notable effects observed in the United States and the United Kingdom. In contrast, South Korea and Japan experienced relatively minimal effects. The reduction in output caused by the pandemic has affected major economies in multiple sectors, including real industries such as forestry and fisheries, and the services such as hotels and restaurants.

**Conclusion:**

The overall negative macroeconomic impact of the epidemic on major economies has been significant. Strategic interventions encompassing initiatives like augmenting capital supply, diminishing corporate taxes and fees, offering individual subsidies, and nurturing international cooperation held the potential to mitigate the detrimental economic consequences and enhance the global-economic amid the pan-demic. Consequently, this study contributes to the advancement of global anti-epidemic policies targeting economic recovery. Moreover, using the CGE-COVID-19 model has enriched the exploration of general equilibrium models in PHEIC events.

## 1 Introduction

During the Spring Festival in 2020, COVID-19 pandemic broke out, and the number of confirmed cases in 1 month quickly surpassed that of SARS in 2003. The World Health Organization (WHO) declared the outbreak a Public Health Emergency of International Concern (PHEIC) due to its speed, infectivity and difficulty in prevention and control. However, the epidemic has since spread rapidly worldwide, and the strain has continued to mutate. Countries worldwide have continued to explore ways to prevent the spread of the epidemic, with some countries adopting measures such as “home quarantine” and “restrictions on entry and exit” (1, 2). Most countries implemented or extended various preventive measures as the epidemic raged. COVID-19 pandemic caused significant damage to people’s health (3, 4) and dealt a massive blow to the world economy (5, 6).

In 2022, the advent of the more transmissible Delta and Omicron COVID-19 variants precipitated a surge in global infection rates, intensifying the struggle to control the pandemic. This health crisis, compounded by the Russia-Ukraine conflict, has triggered escalating food and energy inflation. Consequently, the international trade context and economic stability are declining, particularly as the pandemic persists in the Asia-Pacific, Europe, and North America, burdening supply chains and decelerating economic growth. Despite efforts to reinstate the international economic order, it confronts both internal and external tribulations: domestic markets languish, consumer spending is tepid, and import growth remains stunted; simultaneously, diminishing external demand is resulting in substantial order losses for export businesses, potentially amplifying systemic economic risks.

By 2023, a semblance of normalcy began to return, heralding the onset of an economic resurgence post-COVID-19. However, the future of the international economy continues to be fraught with uncertainty, with ongoing disruptions to industrial supply chains stymieing economic recovery. Presently, the JN.1 COVID-19 variant has been detected in 12 nations. Its proliferation has led health authorities in the UK and the US to brace for a possible pandemic resurgence. On December 19, 2023, the World Health Organization issued a preliminary risk assessment, classifying JN.1 as a “Variant of Concern.”

The COVID-19 pandemic has profoundly affected economic structures, social employment, supply chains, and financial systems, with a recovery trajectory that is highly unpredictable. In response, nations worldwide have enacted a battery of monetary and fiscal policies, including quantitative easing, to bolster consumption and mitigate the pandemic’s detrimental impact. Consequently, evaluating the effects of these policies has become a pressing concern for policymakers and scholars alike. This paper introduces an innovative model designed to assess policy effectiveness and to inform the refinement of economic recovery strategies.

## 2 Review of the literature

Research on the economic impact of PHEIC, particularly epidemic diseases, has focused on healthcare costs, focusing on both direct expenses (such as public health resourcing and treatment costs) and indirect costs (such as production impacts due to labor losses due to work stoppages) associated with the disease (7, 8). Sands et al. argue that the assessment of the economic risk of an epidemic should take complete account of the evaluation of the risk of the disease to the economic system (9). Brahmbhatt and Dutta equally argue that even if the chances of illness and death from some infectious diseases such as SARS are slight, the uncoordinated, panicked prevention and control measures taken to avoid infection could cause significant economic damage (10).

In response to the impact of epidemics (such as SARS, H1N1, and so on) on the economic system, several scholars have quantified the economic impact of epidemics on various countries and regions of the world. Ridel et al. found that social factors such as the growth of international trade and the transfer of large amounts of labor across borders contributed to the spread of epidemics and infections (11). Dixon et al. evaluated the impact of the H1N1 pandemic on the US economy with a CGE (Computable General Equilibrium) model (12). They found that the impact of the influenza peak on demand (such as reduced international travel and leisure activities) for US tourism was noticeably more significant than the impact of supply (such as reduced productivity). Keogh-Brown et al. estimate the hazards of different levels of “pandemic” disease by constructing a UK-France-Belgium-Netherlands multi-country multisectoral CGE model, which finds that school closures and preventive absenteeism double the potential economic costs impact in these countries, and that prudent prevention and control plans can help mitigate high policy costs (13). Lee and McKibbin used the G-Cubed model to assess the economic impact of SARS on nine Asia-Pacific countries and regions, including China. They concluded that the economic impact of SARS on countries such as China was mainly in terms of the consumption behavior of households and businesses (14). Orish has found that epidemics such as Cholera and Ebola can worsen poverty in Africa, particularly in sub-Saharan African countries, and have a direct negative impact on the economies of infected countries, thereby reducing economic growth and productivity in these countries (15).

However, some scholars have also focused on individuals’ or governments’ behavioral decisions and choices during epidemics. “SARS-type” effects suggest that outbreaks of infectious diseases have high human and economic costs in terms of illness and death and that even when the chances of eventual disease or the death toll are small, they cause severe economic disruption so that active government policies will have positive expected effects. For example, Brahmbhatt and Dutta conduct a game-theoretic analysis of the economic damage caused by SARS in East Asia in 2003 and the plague in India in 1994 respectively, and show that proactive government action can largely avoid unnecessary economic damage caused by the epidemic (10). Ridel et al. found that the growth of international trade, large cross-border movements of people, and incomplete public health systems all contribute to the spread of infections and epidemics so that countries would prioritize disease surveillance and develop a strategy based on early warning and rapid response mechanisms (11). As for prevention and control research, Meltzer et al. and Prager et al. conducted separate studies on the threat of a potential pandemic influenza outbreak to US industrial operations and the overall economy (8, 16). They both found that proactive prevention and control measures such as increased personnel engagement and government action could save business and personal treatment expense and effectively mitigate net losses in GDP. Jackson et al. also found that the health care and overhead savings from more cost-effective prevention strategies far exceeded the costs of pandemic preparedness and management (17).

COVID-19 pandemic went from being the most extensive “Black Swan event”^1^ to the most prominent “Grey Rhino event”,^2^ and its domino effect is increasingly being studied by economists. Fernando and McKibbin estimate economic losses in 24 industrial countries over seven scenarios. The worst-case scenario sees a sharp fall in consumption and investment, leading to a sharp fall in stock prices and a sharp fall in bond profits (18). Hofman’s analysis concludes that COVID-19 pandemic impedes labor mobility, thereby reducing productivity, disrupting supply chains, inhibiting exports, leading to increased uncertainty, and directly causing a further decline in trade and manufacturing growth, severely affecting the world economy (19). Appleby argues that while fiscal subsidies and loose monetary policies implemented by many governments, especially those of large countries, will increase the fiscal burden on countries and bring about global inflation, attempts by some countries to initiate new trade frictions on the pretext of epidemic prevention and control will increase the cost of international trade (20). Li et al. conclude that containing the spread of the disease should be prioritized over restoring economic activity by conducting a longitudinal survey of people’s expectations of epidemic control and maintaining positive economic growth (21). In addition, several scholars have explored the macroeconomic or industrial economies of different countries and regions separately, arguing that the epidemic had a significant deterrent effect on economic growth and caused powerful shocks to capital markets, labor markets, and people’s living standards and that the right policy mix could reasonably reduce losses in all areas (22–36).

Reviewing the available literature, it is evident that the following areas need for improvement in the economic impact of epidemics: Upon reviewing the existing literature, it becomes apparent that there are certain deficiencies in understanding the economic impact of epidemics. Firstly, previous international research has predominantly focused on the localized economic consequences of specific epidemics such as H1N1, H5N1, and SARS, neglecting a comprehensive analysis of the macroeconomic and industrial impact on a global scale. Consequently, the broader implications of “pandemic” epidemics on the global economy remain understudied. Secondly, while scholars have offered qualitative insights and recommendations on the effects of COVID-19 pandemic on macroeconomics or specific industries within specific regions, there is a lack of quantitative analyses that encompass a comprehensive evaluation of the global economic system. Furthermore, existing studies often exhibit limitations by narrowly setting parameters for specific aspects, such as demand or trade, within general equilibrium models, which compromises the validity of the evaluation results.

**Figure 1:**
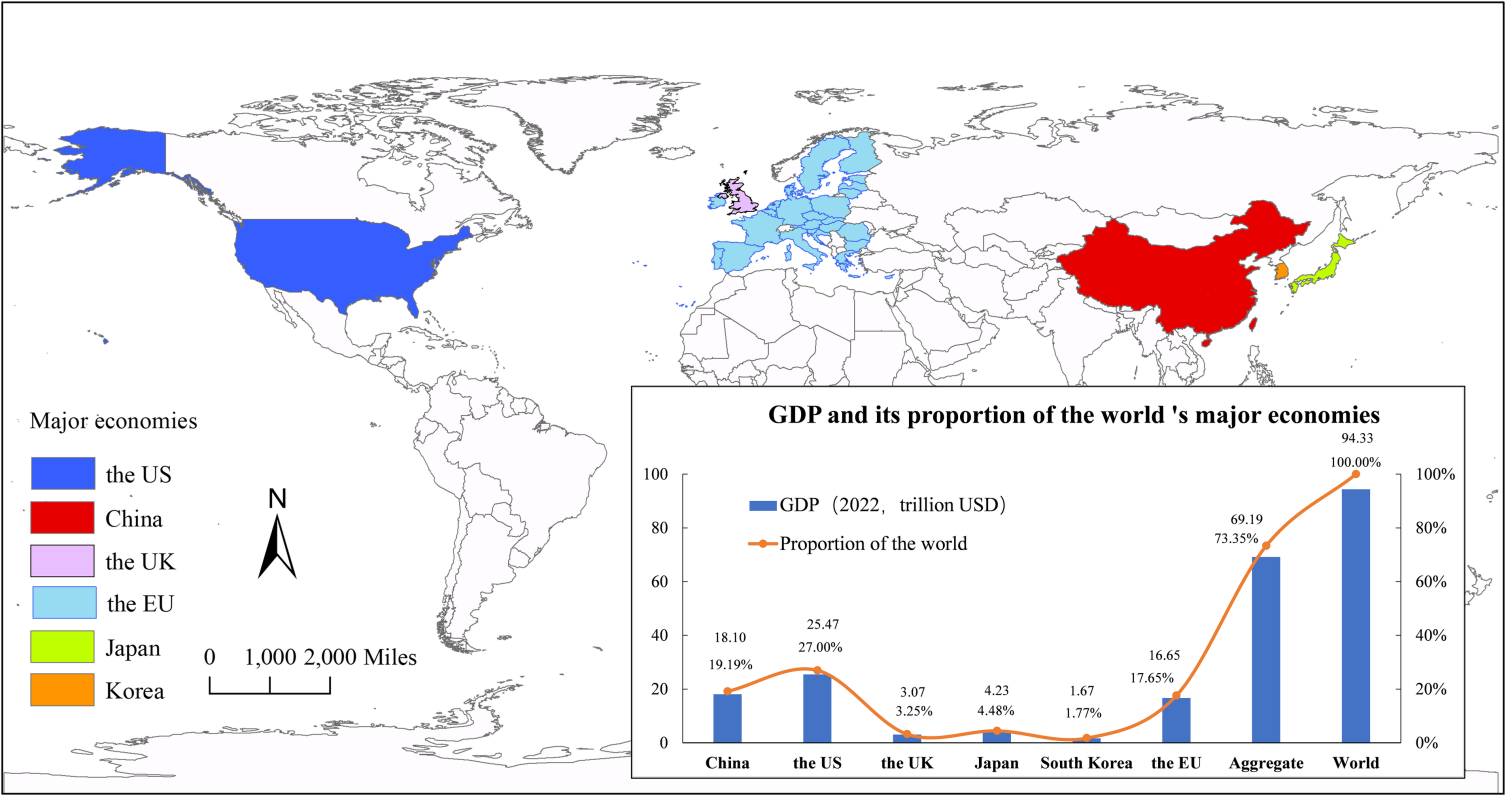
Distribution of the world’s major economies and their economic conditions in the study. The US, China, the UK, the EU, Japan and South Korea are the countries and regions severely affected by the current COVID-19 pandemic outbreak. The combined GDP of these six economies accounts for 70% of the global economy, and substantial economic and trade links exist among these countries.

To address these deficiencies, this study introduces three significant innovations. Firstly, a multi-country, multi-sector CGE-COVID-19 model is constructed to comprehensively assess the macroeconomic and industrial impacts of the New Coronary Pneumonia epidemic on the six major economies: the US, China, the UK, the EU, Japan, and South Korea (Figure 1). This approach enables a thorough understanding of the epidemic’s effects across different sectors and countries. Secondly, the study evaluates the effectiveness of policies implemented by these countries and regions in response to the epidemic. By considering the diverse impacts of COVID-19 pandemic on various economic aspects (such as supply and demand, trade, etc.) and the range of countermeasures employed (such as capital supply, subsidies, etc.), the model parameters are accurately set to provide valuable insights for global economies in formulating policy measures to mitigate the impact of epidemics. Lastly, the utilization of the CGE-COVID-19 model expands the exploration of general equilibrium modeling within the context of Public Health Emergency of International Concern (PHEIC) events, offering a novel and comprehensive approach to studying the economic consequences associated with such events.

After review, we found that most of the studies on COVID-19 epidemic are statistical analyses or review studies, and there is a lack of empirical analysis of relevant models, not to mention nonlinear analysis of various economic indicators. The COVID-19 epidemic has directly caused a slump in international trade and a rise in unemployment, and indirectly affected global Industry sector restructuring, leading to a global recession. Therefore, this paper strives to fill the gaps in the literature by providing a comprehensive analysis of the economic impact of COVID-19 epidemic on a global scale, evaluating policy responses, and utilizing an innovative modeling approach. By addressing these deficiencies, this research aims to contribute to the advancement of knowledge in the field of epidemic economics and assist policymakers in developing effective strategies for economic recovery.

## 3 Theoretical basis

### 3.1 General equilibrium theory and CGE model

Johansen established the CGE model, based on the general equilibrium theory, to evaluate the impact of tax policy changes on the economy (37). After 60 years of refinement and development, the CGE model has been widely used by academics and research institutions evaluating the impact of domestic and international factors on the economy of one or more countries.

**Figure 2:**
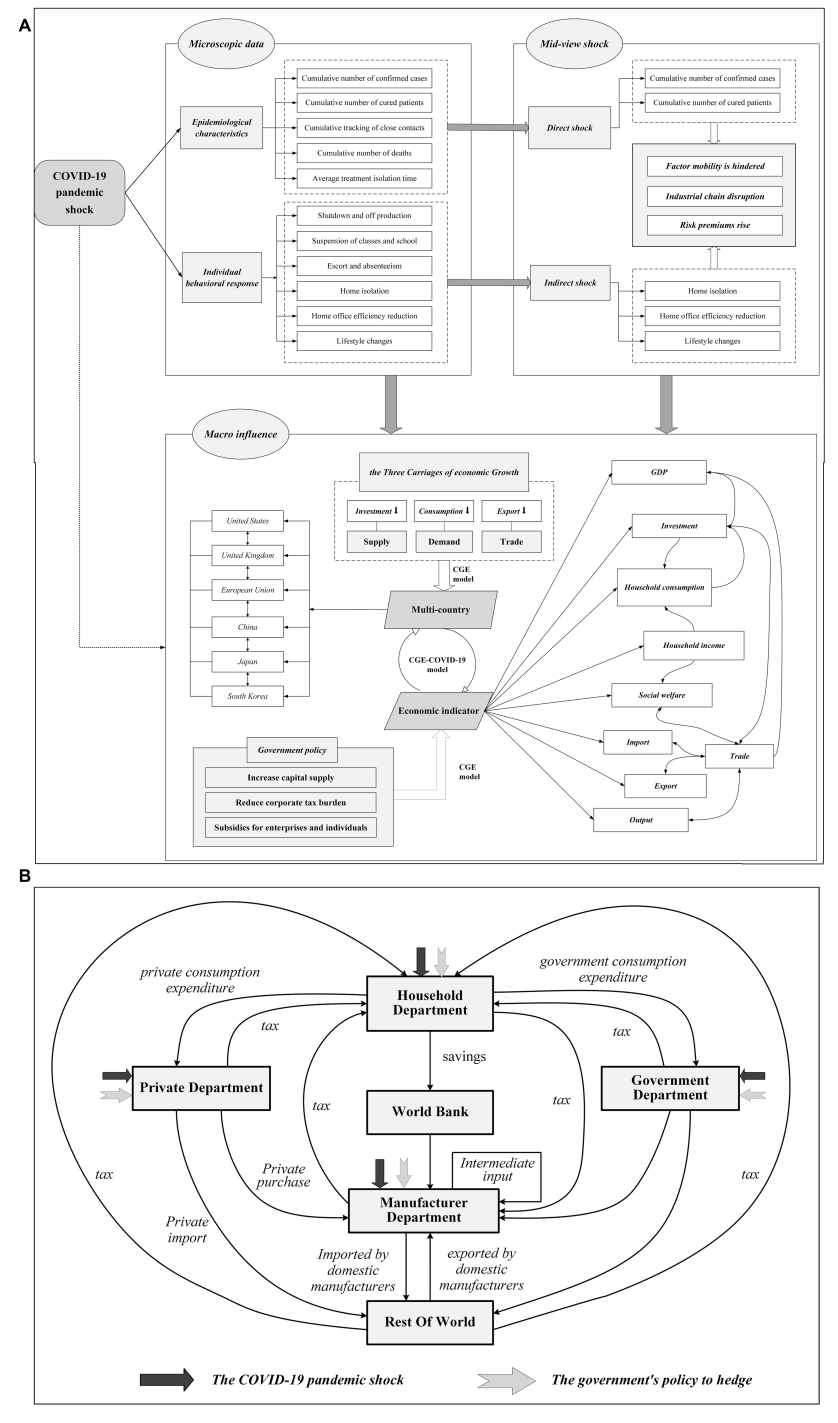
A global CGE model with COVID-19 shocks and government policies. **(A)** Analyzing COVID-19 Economic Impact Micro, Meso, Macro Perspectives, **(B)** CGE-COVID-19 Model.

The CGE model rests on the premise that an economy’s commodities and production factors, when subjected to external shocks under open market conditions, can precipitate adjustments in a nation’s import and export dynamics through the mechanism of international trade. These adjustments potentially trigger a domino effect of economic activities within the domestic economy and induce variations in the prices as well as the supply and demand of goods and production factors internationally. The model posits that these shifts continue until global market transactions reach a new equilibrium where supply aligns with demand, engendering impacts on production, income, consumption, social welfare, and the broader spectrum of investment and trade activities—both domestically and across other economies. A typical global CGE model is depicted in Figure 2. The GTAP (Global Trade Analysis Project) model, which is now widely used by academics, is a multi-country (38), multi-sector global CGE model designed by Prof. Thomas W. Hertel of Purdue in the USA, based on neo-classical economic theory (39).

The GTAP model can analyze the impact of political and economic factors on the macro economy (GDP, population income and consumption, social welfare level, capital return, trade balance, etc.) and industries (output and product prices, etc.) of one or more countries from a global perspective. Therefore, a CGE model can be constructed to assess the impact of the New Coronary Pneumonia outbreak on the economies of the US, China, the UK, the EU, Japan, and South Korea, and to explore the effects of the policies of the above economies in response to the outbreak.

### 3.2 The theoretical logic of the impact of COVID-19 on economic shocks

The underlying theoretical rationale and the mechanism of internal variable transmission for this paper are outlined in Figure 2A. Our primary theoretical foundation revolves around the mutual influences among various indicators. The dynamic mechanism is predicated on two aspects: the economic impact exerted by the COVID-19 pandemic on different economies, and the diverse policy measures these economies have deployed to counteract the pandemic’s adverse effects.

Drawing from a literature review, we understand the pandemic’s impact in terms of direct and indirect effects. Ultimately, these effects resonate across macro, micro, and meso-economic levels.

From a microdata perspective, the factors at play include epidemiological characteristics and individual behavioral responses. Epidemiologically, factors such as confirmed cases, recoveries, deaths, and quarantine are responsible for direct effects. Indirect effects emerge from shutdowns, caregiving, absenteeism due to quarantine, and lifestyle changes, all of which disrupt economic activities. These aspects, at the meso level, lead to hindered element flows, supply chain fractures, and increased risk premiums, inflicting indelible damage on macroeconomic influence. This, in turn, affects the three pillars of economic growth: investment, consumption, and exports, corresponding to the supply side, demand side, and trade aspect scenarios set out in our study.

At the macro level, the CGE-COVID-19 model sits at the core of our impact transmission mechanism. As depicted in Figure 2B, this model is a quintessential global CGE framework. It envelops the six major world economies and the variations in their economic indicators.

### 3.3 Macroeconomic closure

**Figure 3:**
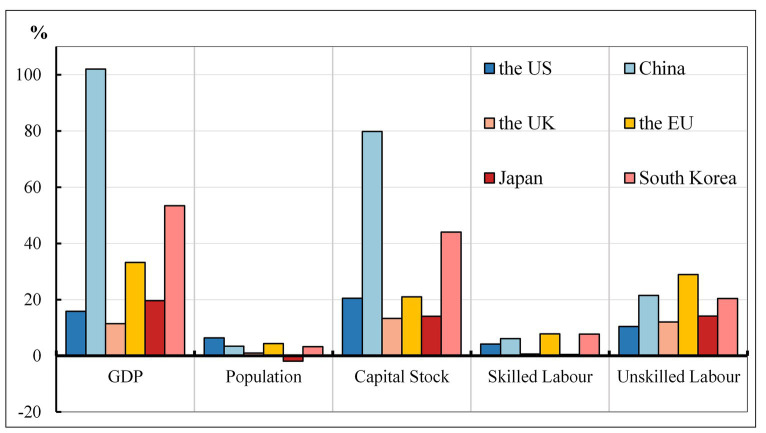
Growth rates of macro variables (2014–2022; %). Source: GDP and population data from IMF, capital and labor force data from CEPII global forecasts.

The GTAP10 database, which is anchored to the year 2014, has been updated to facilitate an accurate analysis of the COVID-19 pandemic’s impact on the economies of the US, China, the UK, the EU, Japan, and South Korea. For the purposes of the CGE-COVID-19 model, the database now includes 2022 data on population, GDP, capital stock, and trade for each of the aforementioned regions. This update utilizes the dynamic recursive approach as outlined by Walmsley et al. (40), which integrates technology variables with GDP variables within the macroeconomic closure of the baseline scenario. Consequently, the model database incorporates exogenously specified economic indicators (GDP), capital stock, demographic data, and labor force composition (divided into skilled and unskilled labor) for each country or region, as well as other macroeconomic data, projected recursively to the year 2022 as illustrated in Figure 3.

To align with the model’s short-term closure requirements, the original 65 industry sectors contained within the database have been consolidated into 24 aggregated sectors. To streamline the presentation of this paper, these sectors are denoted in Table 1 as S1 through S24, and this numerical labeling is consistently used in lieu of sector names in all subsequent figures.

**Table 1:** Sector numbers and names.

Sectors	Sectors breakdown details	Sectors	Sectors breakdown details
S1	Cereals and crops	S14	Essential drugs
S2	Fruit and vegetable products	S15	Petrochemical, rubber and plastic products
S3	Oil and sugar crops	S16	Hotel catering industry
S4	Plant fiber	S17	Construction industry
S5	Animal husbandry	S18	Real estate leasing and property
S6	Forestry and fisheries	S19	Traffic communication
S7	Mineral deposits and energy products	S20	Public utility service
S8	Tobacco, alcohol and non-staple food	S21	Retail, wholesale and business activities
S9	Fur and textile clothing	S22	Financial and insurance services
S10	Wood and paper products	S23	Education and health
S11	Transportation and mechanical equipment	S24	Entertainment and leisure
S12	Metals and metal products	OA	Overall level
S13	Electronic equipment		

### 3.4 Scenario setting

#### 3.4.1 Supply (production), demand (consumption), and the trade environment

Given the epidemic’s impact on the economy, the supply side of labor supply was greatly affected by the closure measures. In the short term, businesses ceased production and stopped working, and the movement of the labor force was reduced, all of which had an enormous impact on the labor supply; additionally, as the response policy tended to restrict the movement of people, it also had a significant impact on consumption and trade.

1. The US. As the epidemic repeatedly occurs, a conservative estimate of the average time that the epidemic shuts down production in US businesses is 1 month. The US unemployment rate in March 2022 is 3.611%.^3^ Accordingly, assuming that the number of days of labor supply in the US is halved to 15 days for the year, combined with the reduction in labor supply due to unemployment, the labor supply level is set to fall by 6%.^4^
2. China. Localized outbreaks of epidemics in China have led to the suspension of work, production and schooling in many places, and the inability of the labor force to arrive for average production has caused significant economic losses to society. This paper assumes that the number of days that various frontline labor forces in China cannot typically work due to work stoppages is 10 days, based on the data of the National Bureau of Statistics of China, and adopted the processing methods of Dixon et al. and Zheng et al. (12, 41), the labor supply level is set to fall by 5% in China.
3. The UK and the EU. The actual day of labor supply in the UK are assumed to be reduced by 12 days; the closure measures in the EU countries are essentially 15 days, and due to the better developed manufacturing sector in European countries, this results in an assumed loss of 10 days of actual labor supply in the EU. Likewise, the combined unemployment caused by the epidemic results in a 6% reduction in labor supply in the UK and EU countries.^5^
4. Japan and South Korea. These countries are two indispensable links in the global production chain. The break in the global production chain and the contraction in consumption have forced some companies in Japan and South Korea to cut production due to their heavy reliance on external demand. Thus, it is conservatively assumed that the epidemic caused an actual loss of 1 week in labor supply days in both countries, resulting in a 3% drop in their supply of labor.^6^
5. International Demand and Trade Facilitation. The US is the world’s largest consumer market, and weak demand will directly drag on exports from major trading partners such as China, Canada, Mexico, Japan and Germany. China’s consumption dominates the structure of the economy.^7^ Taking into account of innovative growth due to the epidemic (digital economy), compensatory growth, or government initiatives to exceed expectations with reforms to promote growth, China’s consumption level is set to fall by 5%^8^ for the year and the level of trade facilitation with other countries by 3%.^9^ Economies such as the EU, where the service sector is the main export, suffer heavy losses in the sector, even causing a global service sector crisis. Based on WIOD (2016) data, the average level of cross-border services as a percentage of all industries in the US, UK, EU, Japan and South Korea, which were most seriously affected, was 24.10%, and international tourism expenditure as a percentage of total imports was 19.98%. Based on the number of days considered in the previous section, and considering incentive policies, this paper sets the level of international demand to fall by 5%.^10^ Since the onset of the new epidemic, global consumption levels and trade facilitation levels have been severely affected.^11^ Therefore, the average labor supply and consumption levels in other economies are assumed to decrease by 1%, the level of trade facilitation between countries and regions of the world to fall by 3%, and the international market equilibrium to deteriorate by 0.3%.

In summary, scenario 1 in Table 2 is formed.

**Table 2:** Impact of COVID-19 pandemic on selected countries and response scenarios in 2022.

Scenario setting	Scenario	Scenario descriptions
COVID-19 pandemic shock	Scenario 1	Labor supply levels fell by 6% in the US, 6% in the UK, and 6% in the EU. China’s labor supply levels fell by 5%, Japan’s and South Korea’s labor supply declined by 3% each, and global consumption levels in major economies declined by 5%. Other economies’ average labor supply and consumption levels fell by 2%. The level of trade facilitation between countries and regions fell by 3%, and the balance of international markets deteriorated by 0.3%.
Government Policy response	Scenario 2	China has increased the volume of capital supply by 8%, reduced the corporate tax burden by 12, and 4% subsidy for enterprises and individuals, respectively.
	Scenario 3	The US has increased the volume of capital supply by 10% and the UK by 3%. The EU countries increased capital supply by 5% and introduced a 4% subsidy for businesses and individuals, Japan increased capital supply by 1%, and South Korea introduced a 4% subsidy for household consumption. The rest of the world’s economies increased the capital supply by 2%.
	Scenario 4	In response to the impact of the epidemic (scenario 1), China (scenario 2) and economies around the world (scenario 3) take a variety of effective measures.

#### 3.4.2 Response in China

For a brief presentation, the relevant parameters of the model’s countermeasures are obtained from the policy and measures of each country. Taking China as an example, since 15 May 2022, the Chinese central bank decided to lower the foreign exchange deposit reserve ratio of financial institutions by 1 percentage point, stabilizing the impact of the epidemic on the RMB exchange rate as a result of absorbing the 2020 experience.

1. China increased the capital supply set. The Central Bank will increase the support of prudent monetary policy to the real economy. Thereby setting China to increase capital supply by 8%.^12^
2. China reduces the level of taxation. A shift from the current pattern of tax cuts, mainly for VAT, to a reduction in social security and corporate income tax rates, setting China to reduce its corporate tax burden by 12%.^13^
3. China increases subsidies to businesses and individuals. In 2022, China is assumed to implement a 4% subsidy for both enterprises and individuals.^14^

To summarize, China responds to the epidemic’s impact (scenario 1) by increasing the volume of capital supply by 8%, reducing the corporate tax burden by 12%, and implementing a 4% subsidy for companies and individuals respectively, resulting in scenario 2 in Table 2.

#### 3.4.3 Responses in other regions

Abroad, the epidemic also pushed countries to introduce economic underwriting policies.

1. Assume that quantitative easing monetary policy in the US raises the capital supply by 10%.^15^
2. The UK is assumed to increase the capital supply by 3% in 2022.^16^
3. Assume that the EU countries increase the capital supply by 5% and implement a 4% subsidy for businesses and individuals.^17^
4. Japan is assumed to increase the capital supply by 1%.^18^
5. Set South Korea to subsidize household consumption by 4%.^19^
6. Set the rest of the world economies to increase the capital supply by 2%.

In summary, this leads to scenario 3 in Table 2.

#### 3.4.4 Global response

The scenario setting demands a comprehensive understanding of the interplay between economic indicators, serving as both a critical exposition of the model’s parameters and a logical framework for the study. The COVID-19 pandemic has precipitated a contraction of labor supply within nations. This contraction has a dual impact: firstly, it directly diminishes household incomes through increased unemployment and underemployment, thereby curtailing consumer purchasing power. Secondly, elevated unemployment levels lead to a reduction in enterprise production capacity, lowering total societal output and, consequently, Gross Domestic Product (GDP). Moreover, the pandemic’s effects extend beyond national borders, attenuating domestic production and potentially leading to a decline in the volume and diversity of exported goods, which in turn results in diminished export revenues. Concurrently, a slump in domestic demand may lessen the importation of goods; however, the continued need for essential commodities that cannot be produced locally necessitates sustained importation, potentially leading to a fall in export prices relative to import prices and, ultimately, a deterioration in the terms of trade.

In the face of economic downside uncertainty, economies worldwide should take collaborative measures to address downside risks and seek policy changes to reduce uncertainty. In response to the impact of the epidemic (Scenario 1), both China (Scenario 2) and the world’s economies (Scenario 2) take a variety of practical measures, resulting in Scenario 4 in Table 2.

## 4 Results and discussion

### 4.1 Macroeconomic impact

#### 4.1.1 Assessment of the impact of COVID-19 pandemic on the world’s major economies

1. GDP. COVID-19 pandemic caused a 3.60% decline in China’s GDP. This finding is in line with forecasts by duan et al. (41), which estimates that the epidemic may lower China’s economic growth by 3.5%. COVID-19 pandemic also caused GDP declines in the US, the UK, the EU, Japan and South Korea. Its impact on the macro economy is global (42), and asymmetric across economies (43). In general, the negative impact of the epidemic on the GDP growth of the EU was the largest, followed by the impact on China. In addition, the impact on South Korea, the UK and the US was also significant, and the impact on Japan was the least.
2. Social welfare level. COVID-19 pandemic hurt social welfare levels in all economies, but there were large differences in the magnitude of the changes. The EU experienced the largest decline in social welfare levels at USD564,245 million, followed by the US and China, reaching USD459.240 billion and 404.907 billion USD, respectively. The UK, Japan and South Korea all experienced lower declines in social welfare at less than USD100 billion.
3. Household income and consumption. Consumption is generally considered to be influenced by income and expectations. Expectations of disposable income during the epidemic are the most important driver of expected consumption growth (44). COVID-19 pandemic had a dampening effect on the growth of both household income and consumer spending in all major economies. Specifically, the epidemic may cause the most significant decline in residential income in the EU and China, with a decline of 3.84 and 3.60%, respectively. In contrast, the decline in the US, the UK and South Korea was around 2.50%, and Japan had the most negligible impact. The epidemic had an immense impact on consumer spending in China, with a decline of 7.38%. It may also reduce consumer spending in the EU, the US, South Korea and the UK by 4.77, 3.15, 3.12 and 3.11% respectively, while it had the least negative impact on consumer spending in Japan, with a decline of 1.32%.
4. Net return on capital. The epidemic increases capital market volatility (45) and divergence of capital returns across sectors (46). The COVID-19 pandemic caused a significant reduction in the return on capital in all countries, especially by 7.10% in China, 6.57% in the US, 6.04% in the EU, 4.88% in the UK and 4.50% in South Korea, respectively, while Japan had the slightest change in net return on capital. Such a situation is detrimental to global investment and may lead to disinvestment and short-term capital flight from these countries.
5. Terms of trade. COVID-19 pandemic improved the terms of trade by 0.22% in China, 0.47% in Japan and 0.09% in South Korea. However, it had a worsening effect on other countries. The EU’s trade terms deteriorated the most, with a decline of 0.20%, while that of the UK and the US worsened by about 0.09%.
6. Import and export. Hayakawa and Mukunoki found significantly negative effects of COVID-19 pandemic on both export and import (47). The share of import and export trade decreased in all six economies during the outbreak of COVID-19 pandemic, which was likely to reduce China’s exports by 3.16% and imports by 6.58%. The possible reason was that the epidemic adversely affected investment and consumption demand in China, and reduced investment and consumption demand. Among the export impaction of other economies under the epidemic, the exports in Japan fell the most, with a decline of 5.13%. In contrast, the UK, South Korea, the EU and the US exports also fell by 3.39, 3.16, 2.58 and 2.30%, respectively. Regarding imports, the US, the UK, the EU, Japan and South Korea experienced negative impacts, with an enormous negative impact on the US, which fell by 6.13%. In addition, Japan’s import decline was the smallest, with a decline of 1.96%, and the import decline of the other four economies ranged from 2.56 to 3.58%.
7. Trade balance. Under the outbreak of COVID-19 pandemic, it would likely reduce the US deficit by USD 114.633 billion, and increase the EU’s trade surplus by USD 57.352 billion, China’s by USD 57.132 billion, and the UK’s by USD 5.733 billion. However, it will likely reduce Japan’s trade surplus by USD 28.955 billion and South Korea’s by USD 5.638 billion.
8. Discussion.

**Figure 4:**
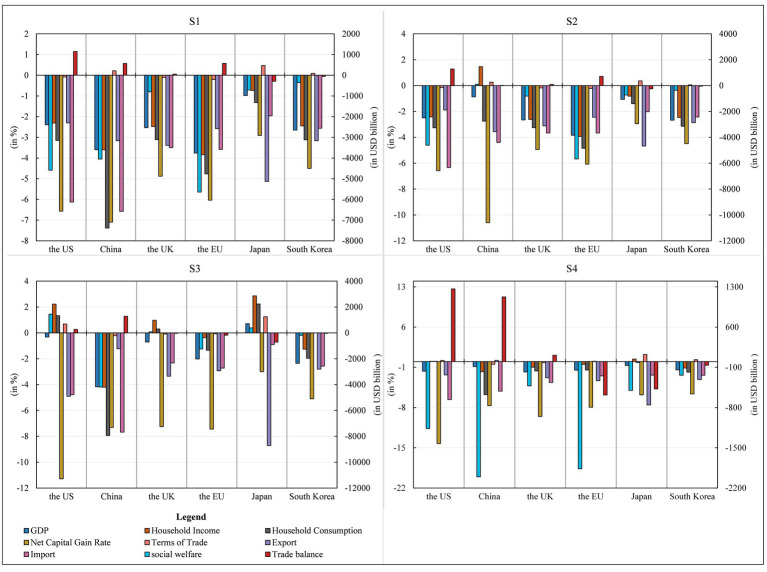
Macroeconomic impact of COVID-19 pandemic on major economies in 2022 GDP, household income, consumer spending, net capital gain rate, terms of trade, export and import (all in %), and the level of social welfare and trade balance (both in USD billion). Source: compiled from CGE-COVID-19 pandemic model results.

Firstly, the analysis of macroeconomic indicators is in Figure 4 shows that the Novel Coronavirus outbreak has harmed all economies. Combining the changes in GDP, social welfare levels, household income and consumption expenditure, and net capital gains, China and the EU suffered the most significantly from the epidemic shock, with all macroeconomic indicators falling at the top of the list; the US and the UK were affected to a lesser extent than China and the EU; and South Korea and Japan were affected the least. The decline in GDP, as noted in the study, is not isolated but intricately linked to a substantial reduction in social welfare levels. This correlation underscores the direct implications of the economic downturn caused by the pandemic on the overall well-being of the population. Furthermore, the dampening effect on household income and consumer spending, particularly pronounced in the EU and China, aligns seamlessly with observed declines in GDP, highlighting a direct relationship between household financial health and a country’s economic performance during a crisis.

Secondly, the significant reduction in the net return on capital across countries signifies increased capital market volatility, reflecting the reported divergence of capital returns across sectors during the pandemic. This dynamic underscores the challenges faced by investors and raises concerns about potential disinvestment and capital flight. Additionally, the varied impact on terms of trade emphasizes the interconnectedness of economies globally, with some countries experiencing improvements while others face deteriorations. This underscores the importance of a nuanced understanding of international trade dynamics during crises.

Thirdly, the negative effects of the pandemic on both export and import, coupled with changes in trade balances, further highlight the interconnected nature of global trade. The disruptions in supply chains and reduced demand contribute to a synchronized decline in both exports and imports across economies. Importantly, the study’s findings reveal global economic disparities, with the EU and China being more severely affected than the US and the UK. These varying degrees of resilience and vulnerability underscore the need for tailored economic policies and recovery strategies.

Finally, a holistic interpretation of the interconnected dynamics among these economic indicators significantly enhances the study’s credibility and applicability. It provides a more nuanced understanding of the intricate relationships shaping the global economic landscape in the aftermath of the COVID-19 pandemic, offering valuable insights for policymakers, economists, and stakeholders navigating the complexities of post-crisis recovery.

#### 4.1.2 Economic effects of China’s countermeasures

Different responses produce different effects (48–52). The government’s strong guarantee policies stimulate economic recovery (53). Appropriate policy responses are necessary (54–58).

1. China’s unilateral response to the epidemic would result in a 0.87% drop in China’s GDP. It would raise China’s social welfare levels by USD 10,381 million, as well as a potential 1.47% increase in China’s income and a 2.75% drop in China’s consumer expenditure. China’s unilateral response to the epidemic presents a nuanced trade-off between economic growth and the effectiveness of countermeasures, resulting in a 0.87% decrease in GDP and 10,381 million emphasizes the impact on the well-being of the population. Concurrently, the positive correlation between government guarantee policies and an increase in social welfare levels by USD. The potential 1.47% increase in China’s income suggests complex linkages between government responses and the financial prosperity of residents. Simultaneously, the 2.75% drop in consumer expenditure reflects the intricate interplay between stimulus measures and individual spending behavior, requiring a nuanced examination.
2. This scenario could also reduce China’s net return on capital by 10.60% and improve the terms of trade by 0.27%. This was mainly because China’s export fell by 3.56% and its import fell by 4.39%, significantly improving compared with the import share in Scenario 1. The 10.60% reduction in China’s net return on capital signals challenges in maintaining profitability amid the crisis, highlighting the delicate balance between recovery measures and a conducive environment for private investment. The 0.27% improvement in China’s terms of trade, coupled with changes in export and import percentages, underscores the intricate relationship between countermeasures and global trade dynamics.
3. China’s response had a positive effect on China’s investment and consumption demand, which led to an increase in imports. While China experiences positive outcomes, including improved social welfare, income, and trade balance, the impact on other economies is less optimistic. Decreases in the US deficit and increases in the EU and UK trade surpluses underscore the complex global economic interdependencies, emphasizing the far-reaching consequences of economic measures undertaken by one country.
4. In addition, an increase in China’s trade surplus of USD 1,425 million significantly reduced China’s trade surplus compared to Scenario 1 (no measures), contributing to a reduction in trade frictions. For other economies, the scenario could reduce the US deficit by USD 128,450 million and increase the EU and UK trade surpluses by USD 70,854 million and USD 9,056 million, respectively. China’s countermeasures led to an improvement in China’s terms of trade, social welfare levels, and residents’ income, while also resulting in a reduction in its surplus. However, the impact on other economies was less positive and may have even led to a decline in GDP, social welfare levels, residents’ income, consumption, and net capital gains in the US, UK, EU, Japan, and South Korea. Specifically, the US deficit decreased, while the trade surpluses of the UK and EU increased and those of Japan and South Korea decreased.

#### 4.1.3 Economic effects of countermeasures taken by other economies in the world

1. GDP. Measures taken by other economies in the world to deal with the epidemic may increase Japan’s GDP by 0.71% while reducing China’s GDP by 4.17%, which would significantly negatively impact China’s GDP growth. South Korea and the EU had a greater negative impact, with GDP falling by 2.35 and 2.02%, while the UK and the US had a smaller GDP decline.
2. Social welfare level. It may lead to an improvement in the level of social welfare in the US, Japan and the UK, with the US having the best effect, increasing by USD 144.598 billion, Japan by USD 38.530 billion, and the UK by USD 7.936 billion, respectively. However, China, the EU and South Korea experienced a deterioration in the social welfare levels, with China experiencing the largest decline of USD 419.346 billion, followed by the EU with a decline of USD 124.330 billion and South Korea with the smallest decline of USD 19.79 billion.
3. Household income and consumption. The household income of Chinese would decrease by 4.20%, and the consumption and expenditure of Chinese residents would also decrease significantly by 7.94%. It may also have a promoting effect on the income of residents in the US, the UK and Japan, in which the income of residents in Japan increased by 2.86%, the income of residents in the US and the UK increased by 2.22 and 0.97% respectively, while the income of residents in South Korea and the EU declines to various degrees. In addition, this scenario may increase consumer spending in Japan, the US and the UK by 2.23, 1.33 and 0.30%, respectively, while it falls in South Korea and the EU by 1.97 and 1.35%, respectively.
4. Net return on capital. Scenario 3 may reduce the net return on short-term capital by 7.32% in China, 11.29% in the US, and 7.44% in the EU, respectively. In other economies, the net return on short-term capital may fall, with the UK falling by 7.25 percent, South Korea by 5.10 percent, and Japan by 3.0 percent.
5. Terms of trade, Import and export. The terms of trade improved by 1.25 and 0.69% for Japan and the US, remained unchanged for South Korea and worsened for China, the UK and the EU. China’s exports fell 1.23%, while imports fell 7.68%. In other major economies except for China, Japan’s exports fell by 8.71%, while those of the US, the UK, the EU and South Korea fell by 4.91, 3.35, 2.93 and 2.81%, respectively. In addition, the imports of the US, the UK, the EU, Japan and South Korea also have a negative impact, with the US having the most significant decline of 4.78% and Japan having the smallest decline of 0.90%.
6. Trade balance. China’s trade surplus increased by USD 128,658 million, a significant increase compared to Scenarios 1 and 2, which worsened China’s international trade environment. In addition, the US trade deficit would decrease by USD 26.942 billion, while the trade surpluses of Japan, the EU, the UK and South Korea would decrease.
7. Discussion.

Compared to Scenario 1, the policy responses to the epidemic in major economies such as the US, UK, Europe, Japan, and South Korea had contrasting effects in Scenario 2. These responses had a positive impact on indicators like GDP, terms of trade, social welfare levels, residents’ income, and trade balance in the aforementioned six economies. However, the impact on China was more negative, resulting in a significant decline in China’s GDP and a notable increase in its trade surplus. The economic effects of countermeasures reveal a web of intricate relationships between various indicators.

Firstly, the impact on GDP demonstrates a nuanced connection with Social Welfare Level. An increase in GDP in certain economies, such as Japan, is correlated with an improvement in social welfare, indicating a positive relationship between overall economic output and societal well-being. The connection between GDP and Household Income and Consumption is also evident. The rise in GDP, particularly in the US, Japan, and the UK, corresponds with an increase in household income and consumer spending. This underscores the interdependence between macroeconomic indicators and individual financial well-being.

Secondly, net return on capital reveals a complex dynamic between short-term capital returns and GDP. The decline in net returns in China, the US, and the EU suggests that economic policies impacting short-term capital flows have repercussions on the profitability of investments, indicating an intricate link between capital mobility and financial returns.

Thirdly, terms of trade, import and export, and trade balance are intricately connected. The improvement in the terms of trade for Japan and the US is associated with reduced trade deficits and negative impacts on imports. This demonstrates how changes in the balance of trade can influence the terms under which countries engage in international commerce.

Finally, trade balance and household income and consumption are intertwined. The increase in China’s trade surplus corresponds with a potential decrease in trade surpluses for other nations. This shift in trade dynamics can have implications for the income and consumption patterns of households, showcasing the delicate balance between international trade and domestic economic conditions.

#### 4.1.4 Macroeconomic effects of countermeasures adopted by all major world economies

1. GDP. Measures taken by all of the world’s major economies in response to the outbreak could reduce GDP by 1.44–1.81% in the UK, the US, the EU and South Korea, resulting in a 0.86% decline in GDP in China and a 0.68% decline in Japan. Compared to Scenario 1 (COVID-19 pandemic impacts), Scenario 4 boosts GDP growth in all these economies.
2. Social welfare level. It would likely reduce the level of social welfare in China by USD 2006.04 billion, and also more significantly in the EU and the US, by USD 186.638 billion and USD 116.561 billion respectively, with smaller decreases in Japan, the UK and South Korea, and a minor decrease in South Korea.
3. Household income and consumption. More representatively, regarding residents’ income, the US and Japan saw a slight increase of 0.05 and 0.44%, respectively; regarding residents’ consumption expenditure, the US saw a slight increase of 0.05%.
4. Net return on capital. The net rate of return on capital declines in all economies, with the US experiencing the largest decline in the net rate of return on short-term capital at 14.25%, the UK, the EU and China experiencing declines in the net rate of return on short-term capital of 9.55, 7.95 and 7.66% respectively, and Japan and South Korea experiencing the most negligible reductions, but also at 5.80 and 5.63%, respectively.
5. Terms of trade. Scenario 4 is likely to worsen the terms of trade for both China and the EU, with both decreasing by 0.47 and 0.18% respectively; however, the conditions of trade improve for Japan, South Korea, the US and the EU, with 1.23, 0.32, 0.20 and 0.06% improvements, respectively.
6. Import and export. Scenario 4 resulted in an increase of 0.21% in China’s exports and a decrease of 5.15% in imports;. In contrast, other economies’ share of exports and imports still declined. In terms of exports, Japan dropped the most, with a decrease of 7.56%, and exports of the US, the EU, South Korea and the UK were down more significantly, by 2.32–3.31%; in terms of imports, the US dropped the most, by 6.60%, and Japan, the EU, South Korea and the UK saw a significant fall in imports, with a drop of around 2.50%.
7. Trade balance. The trade surplus in China and the UK would increase by USD 112.501 billion and USD 10.960 billion, respectively. Furthermore, the trade surpluses of the EU, Japan and South Korea decreased by USD58.171 billion, USD47.607 billion and USD6.445 billion, respectively.
8. Discussion.

The reduction in GDP across major economies, ranging from 1.44 to 1.81%, directly influences the social welfare level. This decrease in GDP translates into diminished resources for social programs, leading to a substantial drop in social welfare, notably in China, the EU, and the US. Simultaneously, changes in GDP have direct ramifications on household income and consumption patterns. Slight increases in residents’ income and consumption in the US and Japan highlight the interdependence between overall economic stability and individual financial well-being. The decline in GDP also contributes to a global reduction in the net return on capital, showcasing the intricate relationship between economic health and capital market performance.

In addition, the decrease in social welfare levels has implications for household income and consumption. Reduced social welfare potentially leads to decreased disposable income, influencing residents’ spending behavior and reshaping consumption patterns. This intricate linkage emphasizes the broader societal impact of macroeconomic policies. The decline in the net return on capital globally is closely tied to changes in the terms of trade. Economic conditions affecting capital returns also impact the terms on which countries engage in international trade. This, in turn, influences import and export dynamics, with China experiencing increased exports and decreased imports. Other economies witness declines in both exports and imports, showcasing the interconnectedness of international trade networks.

It is important that the shifts in import and export patterns further impact the trade balance. China’s increased exports and decreased imports contribute to a larger trade surplus, while the US and other economies face changes in their respective trade balances. Understanding these dynamic connections is crucial for policymakers and analysts, as changes in one economic indicator can have cascading effects throughout an economy. The complex network of interactions underscores the need for a holistic approach in economic analysis and decision-making.

In conclusion, compared to Scenario 1, 2 and 3, Scenario 4 (policies in which major economies jointly respond to the epidemic) boosts GDP, social welfare levels, household income and consumption expenditure in these countries from an overall perspective. However, it is worth noting that the decline in net capital gains in all countries is greater than in Scenario 1 (when no measures are taken). Therefore, economies should consider adopting a synergistic policy approach to counter the negative macroeconomic impact of the New Coronavirus outbreak.

### 4.2 Industry sector economic impact

#### 4.2.1 Assessment of the impact of COVID-19 pandemic on the world’s industry economies

**Figure 5:**
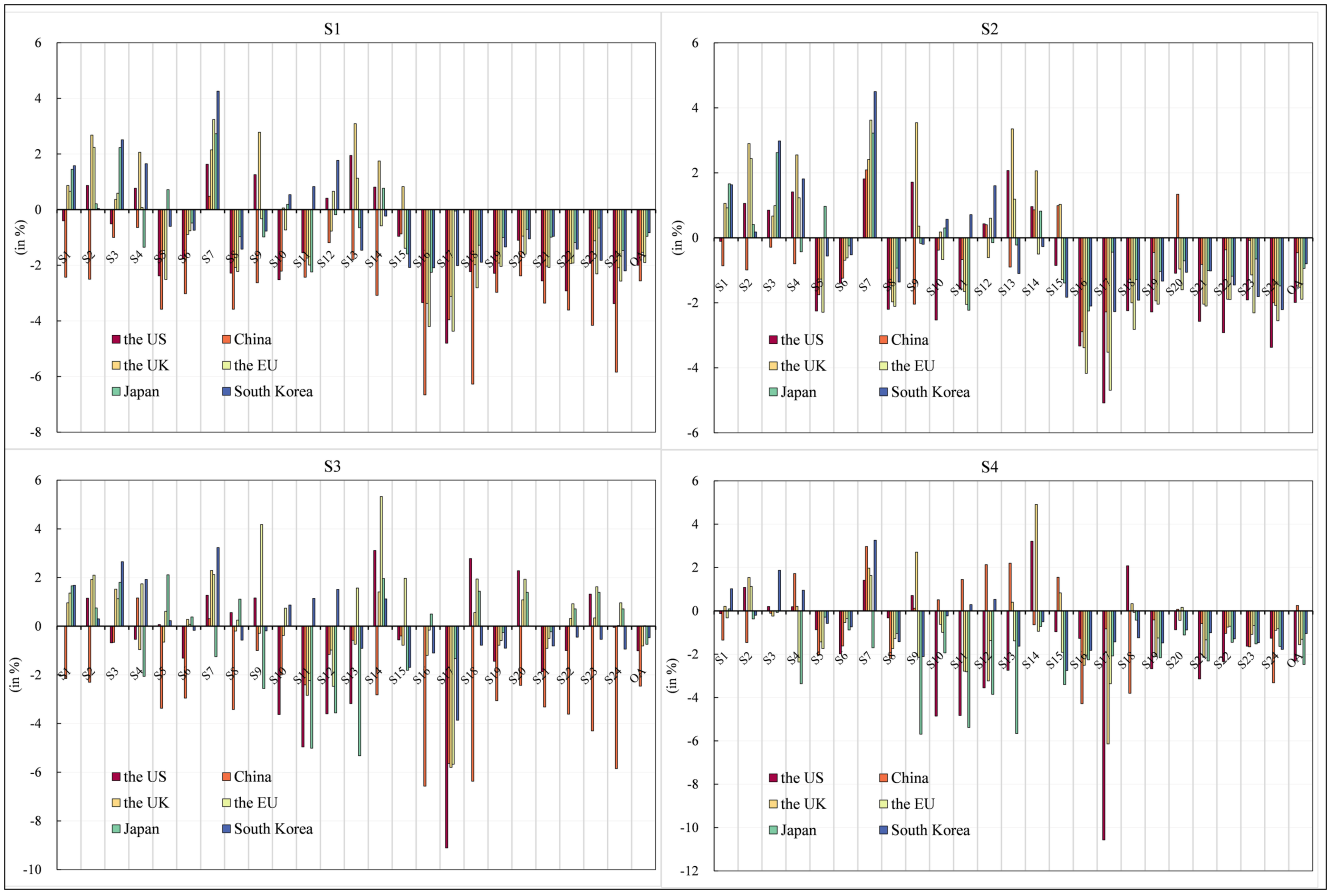
Impact of COVID-19 pandemic shock on sectors in 2022 (%). Source: compiled from CGE-COVID-19 model results.

It is important to recognize the impact of COVID-19 pandemic on the structure of the economy (59). The lockdown policy has spillover effects (60, 61), especially in the food industry, the real estate activities, the constructions and the general services (62). The CGE-COVID-19 model was applied to calculate the impact of the pandemic on the output level of each sector in the world’s major economies in Figure 5.

1. The US and China. The total output level of the US decreased by 2% during the outbreak of COVID-19 pandemic. From the perspective of various sectors, only seven showed positive output growth, while the output level of the remaining 17 sectors all showed varying degrees of decline. Among them, the output level of the construction industry fell the most, by 4.8%. The production level of service sectors such as recreation and leisure, hotel and catering, financial and insurance services, retail, wholesale and commercial activities also declined significantly, ranging from 2.56 to 3.38%. The total output of China decreased by 2.56%. From the perspective of sectors, only the output of mineral deposits and energy products increased slightly by 0.48%, while the output of the other 23 Industry sector sectors declined significantly. This is largely in line with Kekeç et al., who found that the Turkish mining industry was affected to some extent by COVID-19 pandemic, but recovered quickly (63). Service sectors such as real estate leasing and property, recreation and leisure, education and health, construction, and financial and insurance services were hit hard, with output levels likely to fall by 3.61 to 6.27%. The reason is that these sectors belong to the tertiary industry, which is most affected by the decline in consumer demand and employment demand.
2. the EK and the EU. The total output level of the UK fell by 1.53%, with the output level of the hotel and catering industry falling the most by 3.37%. In addition, the output level of construction, entertainment and leisure, retail, wholesale and commercial activities, real estate leasing and property, financial and insurance services, transportation and communication, and other service sectors also declined significantly, with a decrease of 1.92 to 3.12%. The total output of the EU decreased by 1.9%. COVID-19 pandemic caused the output of 17 sectors in the EU to decline to various degrees, among which the output of construction, hotel and catering industry, real estate leasing and property, entertainment and leisure, education and health, retail, wholesale and business activities, transportation and communication and other services declined significantly. Its decline was in the range of 2.04 to 4.37%.
3. Japan and South Korea. The total output level in Japan decreased by 0.96%, with a significant decline in the output of services such as hotels and catering, entertainment and leisure, real estate leasing and property, financial and insurance services, transportation and communication, retail, wholesale and commercial activities. Among them, the output of hotels and catering decreased by 2.25%. The decline in the output of real economic sectors such as transportation and machinery equipment, petrochemical, rubber and plastic products, and plant fibers was also relatively evident. The total output of South Korea decreased by 0.83%. Regarding sector changes, the service industry was the most affected. The top five industries with output impact were recreation and leisure, the hotel and catering industry, the construction industry, real estate leasing and property, and education and health.

The epidemic has an average negative impact on the total output of major economies, especially China, followed by the US, the UK and the European Union. In contrast, Japan and South Korea have a more negligible impact. In terms of the changes in various sectors, the epidemic has reduced the output of forestry, fishery, tobacco, alcohol, non-staple food and other real sectors in major economies, as well as hotel and catering industries, construction industry, real estate leasing and property, transportation and communications, public utility services, retail and wholesale and business activities, financial and insurance services, education, health, culture, entertainment and leisure. In terms of the service sector, which has a sizeable general impact, the epidemic has the greatest negative impact on China, followed by the US, and the most negligible impact on Japan. Specifically, the hotel and catering industry experienced the most significant decline in China, the UK and Japan, while real estate leasing and property output declined the most in China.

#### 4.2.2 Economic effects of China’s countermeasures

1. The US. In addition, the total output of the US decreased by 1.99%. In terms of the output change of various sectors in the US, the output of electronic equipment, mineral and energy products, fur and textile and clothing, plant fiber, fruit and vegetable products, basic pharmaceuticals, oil and sugar crops, metals and metal products increased, among which the output of electronic equipment increased by an enormous amount of 2.07%. However, the decline in construction, recreation and leisure, hotels and restaurants, financial and insurance services, retail, wholesale and business activities, transportation and communication, and other service sectors was still significant.
2. China. Under China’s unilateral measures to deal with the epidemic, the level of China’s total output decreased by 0.46%, among which mineral resources and energy products, petrochemical rubber and plastics, basic medicines, metals and metal products, and utility services increased slightly. Among them, the output of mineral resources and energy products increased by 2.09%, but output in the remaining 19 sectors still fell.
3. The UK and the EU. The total output of the EU decreased by 1.89%. In contrast, the output of the entire economic sectors, such as mineral and energy products, fruit and vegetable products, plant fibers, electronic equipment, oil and sugar crops, cereals and crops, metal and metal products, fur and textile and clothing increased. Among them, the output of mineral and energy products increased by the most (3.62%). However, the other 16 sectors’ output declined to various degrees. The total output of the UK decreased by 1.52%. Except for the physical sectors such as fur and textile and clothing, electronic equipment, fruit and vegetable products, plant fibers, mineral and energy products, basic medicines, grains and crops, petrochemical, rubber and plastic products, oil and sugar crops, wood products and paper products, the output of the remaining 14 sectors all declined to various degrees. The output of the service sectors such as construction, hotel and catering, entertainment and leisure, retail, wholesale and business activities, real estate leasing and property fell significantly.
4. Japan and South Korea. Japan’s total output level fell 0.94%, with the output growth in seven sectors: mineral deposits and energy products, oil and sugar crops, cereals and crops, animal husbandry, essential medicines, fruit and vegetable products, wood products and paper products. It also led to a decrease of 0.80% in South Korea’s total output level. Regarding the changes in the output levels of various production sectors, the output of the entire economic sectors, such as mineral resources and energy products, oil and sugar crops, plant fibers, cereals and crops, and metals and metal products increased slightly. Among them, the output of mineral resources and energy products increased by 4.50%. The output of the other 16 sectors declined, among which, the output of the service sectors such as construction, entertainment and leisure, hotel and catering, real estate leasing and property declined significantly, with a decline in the range of 1.02 to 2.27%.

The various measures taken by China in response to the outbreak were able to significantly mitigate the impact of the Newcastle pneumonia outbreak on China’s total output level. However, the impact on the other five economies’ total output levels was insignificant.

#### 4.2.3 Economic effects of countermeasures taken by other economies in the world

As seen in Figure 5, the response measures taken by economies other than China had little impact on China’s Industry sector sector’s output level. However, they were able to significantly mitigate the impact of the epidemic shock in the US, UK, EU, Japan and South Korea.

1. The US. Responses from economies other than China could result in a potential 1.01% decline in total US output. By sector, output levels are likely to rise in essential medicines, mineral and energy products, furs and textile clothing, fruit and vegetable products, tobacco and alcohol by-products, livestock, real estate rental and property, utility services, and education and health, with cereal and crop output unchanged, but output in 14 other sectors is likely to fall.
2. China. China’s total output declined by 2.46%, with the output of plant fiber, minerals and energy products rising by 1.16 and 0.31%, respectively. At the same time, the remaining 22 sectors show a decline in output, with the service sectors of hotels and restaurants, real estate rental and property, recreation and leisure, construction, education and health, and financial and insurance services showing a more pronounced decline in output levels. However, comparing Scenario 2, it can be seen that adopting policies to deal with the epidemic in the US, Japan, the UK, the EU and South Korea had a minor impact on China’s output.
3. The UK and the EU. The EU total output level fell by 0.81%, with notable rises in output in the real economy sectors of minerals and energy products, fruit and vegetable products, oil and sugar crops, essential medicines, cereals and crops, forestry and fishing, with the most impressive growth of 2.29% in the output of minerals and energy products. In contrast, output in the other 13 sectors rose by varying degrees. The level of total UK output rose by 0.02%. Looking at the sectors, except construction, transport and communications, retail, wholesale and business activities, hotels and restaurants, metals and metal products, and transport and machinery equipment, which registered declines, construction saw the largest decrease of 5.67%, while metals and metal products and transport and machinery equipment also saw more pronounced declines; the rest of the sectors saw an increased output, with basic pharmaceuticals production topping the list with a 5.33% increase.
4. Japan and South Korea. Japan’s total output level fell by 0.74%. From a sectoral perspective, except for livestock, basic pharmaceuticals, oil and sugar crops, cereals and crops and other physical sector output rose by 1.66–2.11%, real estate rental and property, utility services, education and health and other services sector output level rose by about 1.40%; however, electronic equipment, transport and machinery equipment, metal and metal products, plant fibers and other physical sector output level fell by a still more prominent, in the range of 2.06 to 5.32%. Total output in South Korea fell by 0.47%. The output levels of the real sectors of petrochemicals and rubber and plastic products, electronic equipment, tobacco and alcoholic beverages, furs and textiles and clothing, forestry and fisheries, and all services declined. In contrast, the output of the real sectors of minerals and energy products, oil and sugar crops, and cereals and crops showed an enormous increase of 1.68–3.23%.

#### 4.2.4 Macroeconomic effects of countermeasures adopted by all major world economies

1. The US. Total output in the US fell 2.31% as other major economies took measures to deal with the impact of the pandemic. The output of basic drugs, mineral and energy products, fruit and vegetable products, fur and textile and clothing, oil and sugar crops, plant fibers, real estate leasing and property management sectors increased, among which the output of basic drugs increased the most by 3.21%. The other 17 sectors’ output fell to varying degrees, with construction recording the largest decline of 10.57%. Compared with Scenario 1, the total output level of the US decreased further under Scenario 4, which may be related to the fact that the economic growth of the US mainly relies on net exports and inventories, and some economic stimulus measures are long-term mechanisms that can backfire in the short term.
2. China. In this scenario, China’s total output level increases by 0.25%. In terms of the various departments, the output of mineral and energy products, electronic equipment, metal and metal products, plant fibers, petrochemical, rubber and plastic products, transportation and machinery and equipment, wood products and paper products, fur and textile and clothing, public utility services increased, especially the output of mineral and energy products increased by 2.97%. This indicated that if the world’s major economies took anti-epidemic measures, they could effectively mitigate the adverse impact of COVID-19 pandemic on China’s output. Under Scenario 4, China’s output levels improve across all sectors, resulting in a 0.25% increase in total domestic output. The change in output across sectors shows an increase in the output level in minerals and energy products, electronic equipment, and metals and metal products, with the output of minerals and energy products, in particular, increasing by 2.97%. However, the scenario works less well for the other five developed economies, which may be related to the relatively low value-added of Chinese products and the greater scope for development.
3. The UK and the EU. The total output level of the EU may decrease by 1.31%. In addition to mineral and energy products, fruit and vegetable products, public utility services and other sectors, which increased slightly by 1.63, 1.13 and 0.16%, the output of the other 21 sectors decreased to varying degrees, among which the output of the construction industry decreased the most, by 3.36%. This scenario can mitigate the epidemic’s negative impact by reducing the EU output. Meanwhile, the aggregate output level of the UK fell by 1.56%. The output of basic medicines, fur and textile and clothing, mineral and energy products, fruit and vegetable products, petrochemical, rubber and plastic products, electronic equipment, grains and crops, plant fibers, real estate leasing and property increased, with the output of basic medicines rising by 4.91%. Under Scenario 4, a slight decrease in the level of UK aggregate output compared to Scenario 1. Compared with Scenario 1, the level of the UK aggregate output was likely to decline slightly under Scenario 4.
4. Japan and South Korea. The output level of Japan decreased by 2.47%, in which the output level of all sectors declined except for the weak growth of 0.09% in grain and crops. Among them, the output level of real economic sectors such as fur and textile and clothing, electronic equipment, transportation and mechanical equipment, metal and metal products, petrochemical, rubber and plastic products, and plant fibers declined considerably, with the decline ranging from 3.36 to 5.69%. Under Scenario 4, the total domestic output of South Korea slightly decreased by 1.05%. In terms of the changes in the output level of each sector, the output of the actual economic sectors such as mineral resources and energy products, oil and sugar crops, cereals and crops, plant fibers, metals and metal products, transportation and machinery and equipment increased, among which the output of mineral resources and energy products increased the most, reaching 3.26%. The output of the other 18 sectors declined to various degrees, with petrochemical, rubber and plastic products and fur and textile and clothing falling the most, by 2.75 and 2.11%, respectively. This may be since Japan was greatly affected by the upstream and downstream of the Industry sector chain, and the high degree of foreign trade dependence between the two countries.

### 4.3 Sensitive analysis

**Figure 6:**
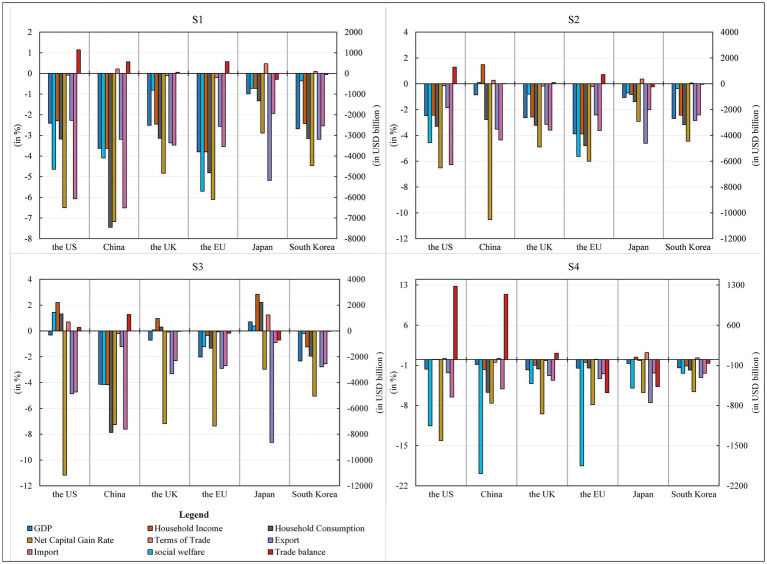
Robustness Test 1—Macroeconomic impact. Source: compiled from CGE-COVID-19 model results.

**Figure 7:**
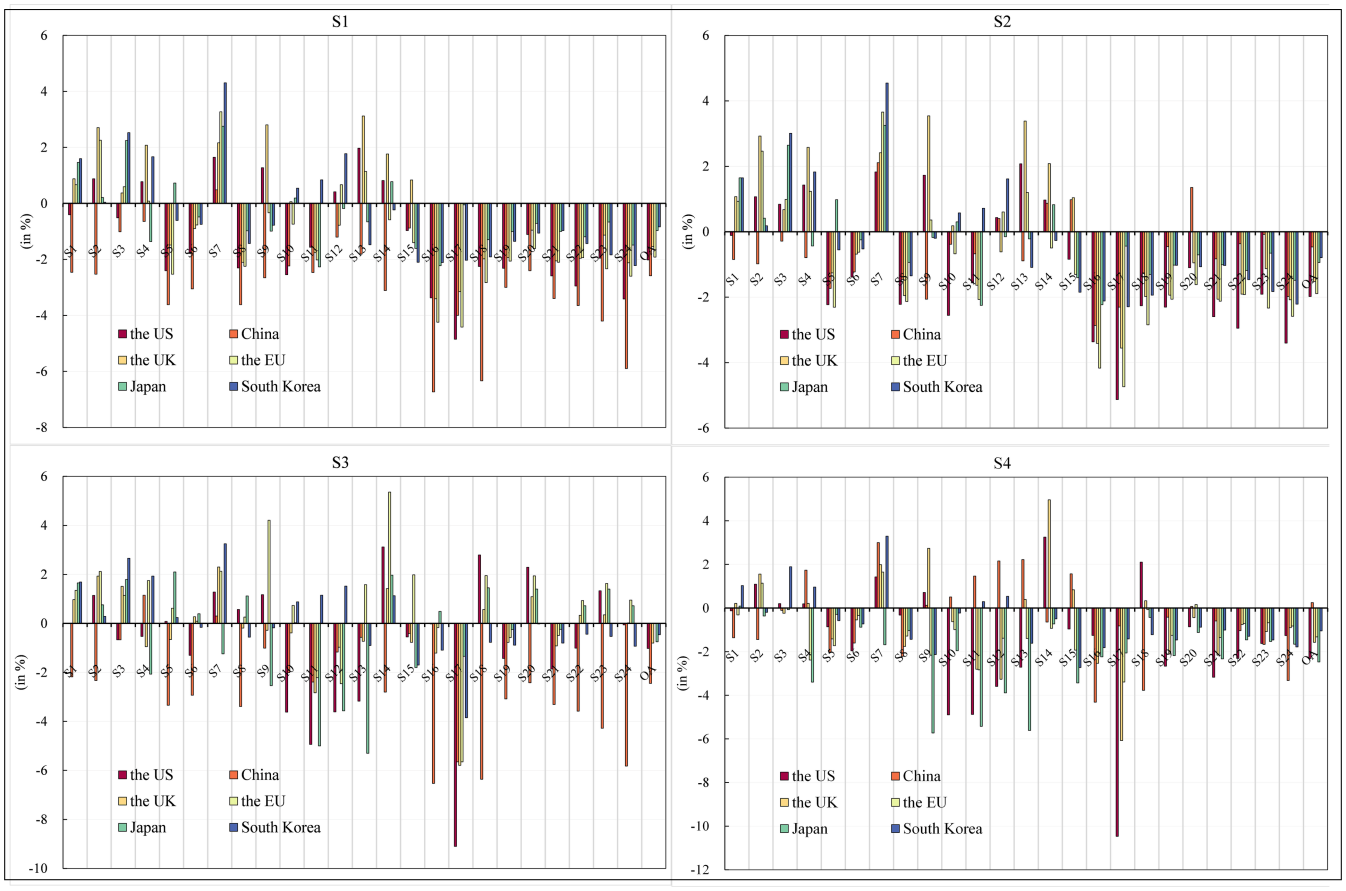
Robustness Test 1—Industry sector economic impact. Source: compiled from CGE-COVID-19 model results.

Considering the results of the above analysis, we conducted two robustness tests. The first sensitivity analysis examined changes in the Armington elasticity of domestic and imported product sources (PSBD) Product Source (PS) and Brand Distribution (BD), Variations in the Elasticity of Product Source (PS) and Brand Distribution (BD) for both Domestic and Imported Products. A higher PSBD means greater substitutability between domestic and imported product sources, and vice versa. To assess the impact of this parameter, PSBD values for product sources were increased by 50%, respectively. As shown in Figures 6, 7, the results of sensitivity analysis show that the numerical difference from the original study is kept within the negligible range of 1%, thus confirming the robustness of the initial conclusion.

**Figure 8:**
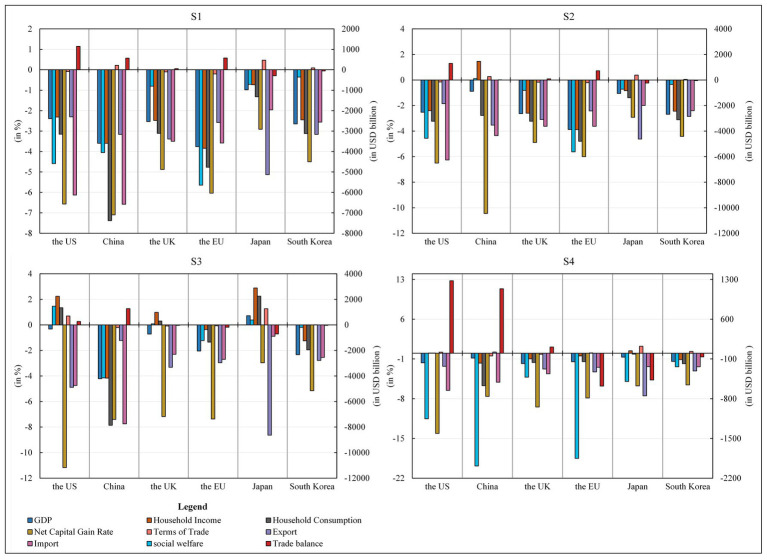
Robustness Test 2—Macroeconomic impact. Source: compiled from CGE-COVID-19 model results.

**Figure 9:**
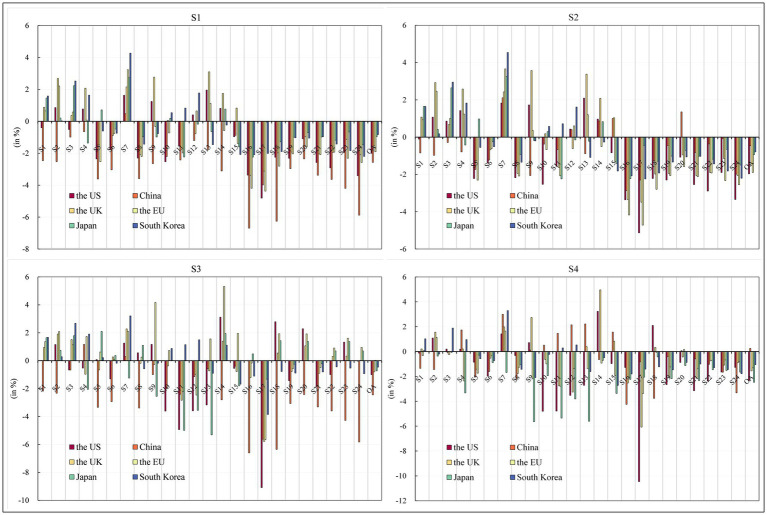
Robustness Test 2—Industry sector economic impact. Source: compiled from CGE-COVID-19 model results.

The second sensitivity analysis examines the effect of changes in the elasticity of Armington on the distribution of product sources in import regions. This parameter is closely related to the trade diversion effect. A higher PSBD suggests that, for the six economies studied in this paper, it is easier to make up for product import shortfalls by importing products from other countries. PSBD values were increased by 50% for each product type. Similar to the first sensitivity analysis, the results show that the numerical difference from the original study results is within the negligible range of 1%, thus reaffirming the robustness of the preliminary conclusions (see Figures 8, 9).

## 5 Conclusion

Based on the CGE-COVID-19 model, this study provides a thorough analysis of the impact of the 2022 new coronavirus pneumonia epidemic on the supply and demand sides of the world’s major economies: China, the US, the UK, the European Union, Japan, and South Korea. This is an expansion of current studies that focus primarily on the impact of the epidemic on individual countries (26, 28). Furthermore, considering the economic consequences of the epidemic, potential countermeasures that may be adopted by China and other countries in response to the crisis are simulated and analyzed. After a comprehensive analysis of the results, the following conclusions can be drawn.

First, the COVID-19 pandemic harmed GDP growth, terms of trade, social welfare level, household income and consumption expenditure, the net return on capital, and import and export of all economies. The COVID-19 pandemic leads to a reduction in the supply of labor in a country. On the one hand, underemployment directly reduce household income and reduce consumers’ purchasing power. On the other hand, the rise of unemployment directly leads to the decline in the production capacity of enter-prises, the reduction of domestic production, the reduction of the quantity and type of export commodities, resulting in a decline in export income. However, if some key commodities cannot be produced domestically, it may be necessary to continue to import, which is prone to the decline of export prices relative to import prices, thus leading to the deterioration of the terms of trade. This is consistent with the majority of scholars finding a significant negative impact of the pandemic on the global economy (41, 50). Based on the changes in GDP, social welfare level, household income and consumption expenditure, and net return on capital, China and the EU suffered the most apparent losses caused by the epidemic’s impact, with all macroeconomic indicators falling at the forefront. The US and the UK have been hit less than China and the EU. In addition, South Korea and Japan were the least affected. Therefore, the overall negative impact of COVID-19 pandemic on the macro economy of major economies was significant and needed to be paid great attention to (42).

Second, since labor is one of the basic elements of production, the reduction in labor supply directly leads to the decline in the productive capacity of many industries and enterprises. Therefore, in the short term, total social output may be affected. COVID-19 pandemic hurt the aggregate output of major economies on average, especially China, followed by the US, the UK and the EU, while it had a negligible impact on Japan and South Korea. However, the impact on different industries is heterogeneous (62). In terms of sectoral changes in output levels in major economies, the similarities were that the pandemic crisis reduced the output of services, and manufacturing sectors (forestry, fishery, real sectors such as tobacco, alcohol and non-staple food, hotels, catering, construction, estate leasing, property, transportation and communication, public utility services, retail and wholesale, commercial activities, financial and insurance services, education and health, entertainment and leisure) (32, 36).

Third, government intervention helps the economy recover (53, 54). China’s policies yielded a positive influence on various aspects, including the terms of trade, the level of social welfare, households income and the trade balance, thus leading to a reduction in the surplus. The proactive measures adopted by China in response to the pandemic crisis played a pivotal role in effectively mitigating its detrimental effects on the GDP (30, 31). These policies include fiscal policy, monetary policy, labor market policy, foreign trade policy, industrial upgrading and structural adjustment. Only when these measures are coordinated and synergistic can they effectively promote economic recovery (64).

This paper thus puts forward the following policy recommendations for countries to alleviate the epidemic: firstly, increase macroeconomic policy support by adopting an active fiscal policy on the one hand and fully applying monetary policy on the other; secondly, appropriately increase enterprise subsidies to address the demand for funds for enterprises to resume work and production; thirdly, provide subsidies to individuals and households to boost domestic demand and consumption (65, 66); fourthly, employment is a matter of national importance and livelihood, and the employment pressure should be kept vigilant. In response to the challenges posed by the COVID-19 pandemic, there is a pressing need to expedite the cultivation and advancement of emerging industries and dynamic energy sources (67, 68). Moreover, urgent attention should be given to the development of crucial infrastructure in sectors such as new energy and healthcare. Additionally, it is imperative to ensure the stability of the existing employment structure and ratios while making concerted efforts to bolster enterprises in their capacity to absorb labor. The last but not the least, establishing a global response mechanism to jointly address the next pandemic.

What needs to be stressed is that the COVID-19 epidemic has had a huge impact on the economy and society in the past 3 years, and will have a profound impact on future economic development. At this stage, it is particularly important for our research to provide data support for governments to adopt practical, scientific and accurate responses to the epidemic. However, there is an unavoidable problem that the epidemic situation changes rapidly, and there may be deviations between the data analysis results and the actual situation due to technical obstacles and difficulties in obtaining data, which should be further explored in future studies.

## Data availability statement

The original contributions presented in the study are included in the article/Supplementary material, further inquiries can be directed to the corresponding author.

## Author contributions

MS: Conceptualization, Methodology, Software, Supervision, Writing – original draft. SY: Data curation, Formal analysis, Investigation, Writing – review & editing. TC: Funding acquisition, Resources, Visualization, Writing – review & editing. JZ: Investigation, Project administration, Formal analysis, Validation, Writing – review & editing.

## Footnotes

^1^“Black Swan event” describes an unexpected, rare, and impactful occurrence that defies conventional predictions and is difficult to anticipate.

^2^“Grey Rhino event” is a likely and visible risk that is often overlooked or underestimated, leading to significant impact.

^3^https://data.worldbank.org.cn/indicator/SL.UEM.TOTL.ZS?view=chart

^4^Manufacturing is the main sector affected (manufacturing and real estate account for 17% of US GDP and the cultural and entertainment industries account for 40%), while other industries such as power generation, telecommunications, communications, education and the internet are still able to work and work online.

^5^In view of the situations of the UK and the EU are similar to the US, for the purpose of calculation, the labor supply level is also set to fall by 6%.

^6^The average working hours in East Asian countries are relatively long, 45 h per week in Japan and 46.5 h per week in South Korea, both of which are similar to China’s level. For the purpose of calculation, the one-day labor supply levels in China, Japan, and South Korea have been equated in this study.

^7^In the eurozone, tourism, catering, aviation and manufacturing are some of the industries that European countries rely on to survive are even more seriously affected, the UK’s annual car production will decline due to the epidemic, European tourism is damaged, tourism workers face great risk of unemployment; Japan and South Korea’s economy relies heavily on exports, the supply chain production in both countries is seriously hampered.

^8^Calculated based on the relevant data of the National Bureau of Statistics of China.

^9^According to the OECD’s “Trade Facilitation Index,” China’s overall ranking is only 51st in 2022, which is 44th in 2019.

^10^Based on WIOD (2016) data, the average level of cross-border services as a percentage of all industries in the US, UK, EU, Japan and South Korea, which were most seriously affected, was 24.10%, and international tourism expenditure as a percentage of total imports was 19.98%. Based on the number of days considered in the previous section, and considering incentive policies, the level of international demand was set to fall by 5%.

^11^A few important facts: global container freight rates continue to climb, with the market price of containers on some routes once soaring to more than 10 times; global port congestion, with dozens of cargo ships still lined up in large ports such as Los Angeles and Long Beach, California, waiting for weeks to unload; and a report by consultancy firm Deloitte showing that more than 80% of industries face supply chain disruptions and 75% of companies are considering withdrawing their factories overseas and setting them up closer to home.

^12^In 2022, the People’s Bank of China will increase the support of prudent monetary policy for the real economy, and increase re-loans of 100 billion yuan to support coal development and use and enhance energy storage; The Chinese government has introduced a series of policies to promote investment, such as nearly 6 trillion yuan of new infrastructure “investment projects.

^13^According to the policy of the State Taxation Administration of China, the part of the annual taxable income of small and low-profit enterprises not exceeding 1 million yuan shall be included in the taxable income at a reduced rate of 12.5%, and the enterprise income tax shall be paid at a tax rate of 20%. For enterprises in need of key state support, the enterprise income tax shall be levied at a reduced rate of 15%.

^14^On March 18, 2020, the Department of Consumption of the Ministry of Commerce of China encouraged qualified regions and enterprises to launch various types of consumption coupons and shopping coupons for specific groups, specific commodities and specific fields; In 2021, from the central to local relevant departments, enrich the effective supply of consumption and stimulate consumer demand, and issue different consumption vouchers.

^15^In early 2021, the President of the US signed the $1.85 trillion American Assistance Program Act. By mid-March 2021, the total fiscal stimulus plate of the two administrations in the US has reached $5.65 trillion, which is 26.4% of 2019 GDP.

^16^On October 27, 2021, UK Chancellor of the Exchequer Rishi Sunak announced the Autumn 2021 Budget Report, which plans to raise an additional 150 billion pounds (about $183.96 billion) in fiscal funding over the next three years.

^17^In April 2021, the European Commission unveiled a debt package called “Next Generation EU,” which aims to raise a total of 800 billion euros over the next 5 years to promote regional economic rejuvenation.

^18^On April 26, 2022, the Japanese Cabinet launched an economic rescue plan with a total fiscal expenditure of 6.2 trillion yen, including a total scale of 13.2 trillion yen including private funds.

^19^On May 12, 2022, South Korea drafted an additional budget of 59.4 trillion won to help the small businesses cope with the pandemic.
